# Metabolism-based approaches for autosomal dominant polycystic kidney disease

**DOI:** 10.3389/fmolb.2023.1126055

**Published:** 2023-02-16

**Authors:** Ivona Bakaj, Alessandro Pocai

**Affiliations:** Cardiovascular and Metabolism, Janssen Research and Development, Spring House, PA, United States

**Keywords:** ADPKD (autosomal dominant polycystic kidney disease), tolvaptan, metabolic reprograming, therapeutic approaches, metabolism & obesity, GLP-1, glucagon

## Abstract

Autosomal Dominant Polycystic Kidney Disease (ADPKD) leads to end stage kidney disease (ESKD) through the development and expansion of multiple cysts throughout the kidney parenchyma. An increase in cyclic adenosine monophosphate (cAMP) plays an important role in generating and maintaining fluid-filled cysts because cAMP activates protein kinase A (PKA) and stimulates epithelial chloride secretion through the cystic fibrosis transmembrane conductance regulator (CFTR). A vasopressin V2 receptor antagonist, Tolvaptan, was recently approved for the treatment of ADPKD patients at high risk of progression. However additional treatments are urgently needed due to the poor tolerability, the unfavorable safety profile, and the high cost of Tolvaptan. In ADPKD kidneys, alterations of multiple metabolic pathways termed metabolic reprogramming has been consistently reported to support the growth of rapidly proliferating cystic cells. Published data suggest that upregulated mTOR and c-Myc repress oxidative metabolism while enhancing glycolytic flux and lactic acid production. mTOR and c-Myc are activated by PKA/MEK/ERK signaling so it is possible that cAMPK/PKA signaling will be upstream regulators of metabolic reprogramming. Novel therapeutics opportunities targeting metabolic reprogramming may avoid or minimize the side effects that are dose limiting in the clinic and improve on the efficacy observed in human ADPKD with Tolvaptan.

## Introduction

Autosomal dominant polycystic kidney disease (ADPKD) is the most common inherited cause of kidney disease with an estimated prevalence between 1:400 and 1:1,000 (Torres et al., 2007; Chow and Ong, 2009; Harris and Torres, 2009). In ADPKD, enlarging fluid-filled cysts develop in both kidneys, eventually leading to kidney failure. Besides kidney cysts that can be very painful, ADPKD can present with extra-renal manifestations such as development of cysts in the liver, pancreas, spleen and epididymis, abnormal heart valves and brain aneurysm (Perrone et al., 2015). Common features of ADPKD are flank and abdominal pain, urinary tract infections, hypertension, and kidney stones (Gabow, 1990; Torres et al., 2007). ADPKD is predominantly caused by mutations in either PKD1 or PKD2 genes encoding for two ciliary proteins, Polycystin 1 (PC1) and Polycystin 2 (PC2) (Harris and Torres, 2009; Takiar and Caplan, 2011). These mutations within epithelial cells of the kidney interfere with multiple pathways located within the cilia and promote proliferation, de-differentiation and fluid secretion resulting in growth of these cells into cysts. Due to the slow progression and the intrafamilial difference in disease severity, it has been suggested that defective clearance of precipitated microcrystals may promote cyst formation and drive kidney injury when Pkd1 or Pkd2 are mutated (Torres et al., 2019a). An increase in cyclic adenosine monophosphate (cAMP) and a simultaneous dysregulation in intracellular calcium in the cystic epithelium seems to play a key role in generating and maintaining fluid-filled cysts (Yamaguchi et al., 2003; Belibi et al., 2004; Di Mise et al., 2018). cAMP activates protein kinase A (PKA) and stimulates epithelial chloride secretion through the cystic fibrosis transmembrane conductance regulator (CFTR) (Sullivan et al., 1998). A vasopressin V2 receptor (V2R) antagonist, tolvaptan, was recently approved to preserve kidney function in ADPKD by lowering vasopressin-mediated cAMP increase (Chebib and Torres 2021). However, considering the potential drawbacks of Tolvaptan i.e., side effects, poor tolerability, and high cost it is important to identify additional pathways and novel therapeutic interventions. Recently, alterations of metabolic pathways (metabolic reprogramming) in ADPKD have shown that the abnormal cystic growth utilize aerobic glycolysis, glutaminolysis and reducing oxidative phosphorylation (OXPHOS) (Padovano et al., 2018; Podrini et al., 2020). This review will review and discuss potential therapeutic approaches targeting metabolism-based pathways in ADPKD.

## Current approved therapies for ADPKD lowering cAMP

### Tolvaptan

Tolvaptan is a vasopressin-2-receptor (V2R) antagonist approved to slow kidney function decline in adult patients with rapidly progressive ADPKD by reducing cAMP levels (Chebib and Torres 2021, Figure 1). Tolvaptan reduces TKV (Total kidney volume TKV, prognostic biomarker for risk assessment in ADPKD, Fick-Brosnahan et al., 2002) and renal function decline. However, its clinical use is limited by poor tolerability due to aquaretic symptoms, potential liver failure and high cost (Chebib and Torres 2021; Müller et al., 2022). Recently Lixivaptan, a selective vasopressin V2 receptor antagonist which was predicted to have a lower risk of hepatotoxicity compared to tolvaptan was discontinued (https://investors.centessa.com/news-releases/news-release-details/centessa-pharmaceuticals-makes-strategic-decision-discontinue). In the kidney, the V2 receptor is mainly expressed in the distal nephron potentially limiting the area of action of V2R antagonists (Mutig et al., 2007; Sparapani et al., 2021). Tolvaptan has been shown to exhibit a partial agonist activity on β-arrestin recruitment whose expression is increased in human ADPKD kidneys (Xu et al., 2018). These data suggest that there is space for safer and tolerated best in class cAMP lowering approaches in ADPKD either targeting additional pathways regulating cAMP (PKA inhibition, PDE activation), biased V2R antagonists or combination with targets that may provide an additive or synergistic effect such as the calcium-sensing receptor (Di Mise et al., 2021; Zhou and Torres, 2022).

**FIGURE 1:**
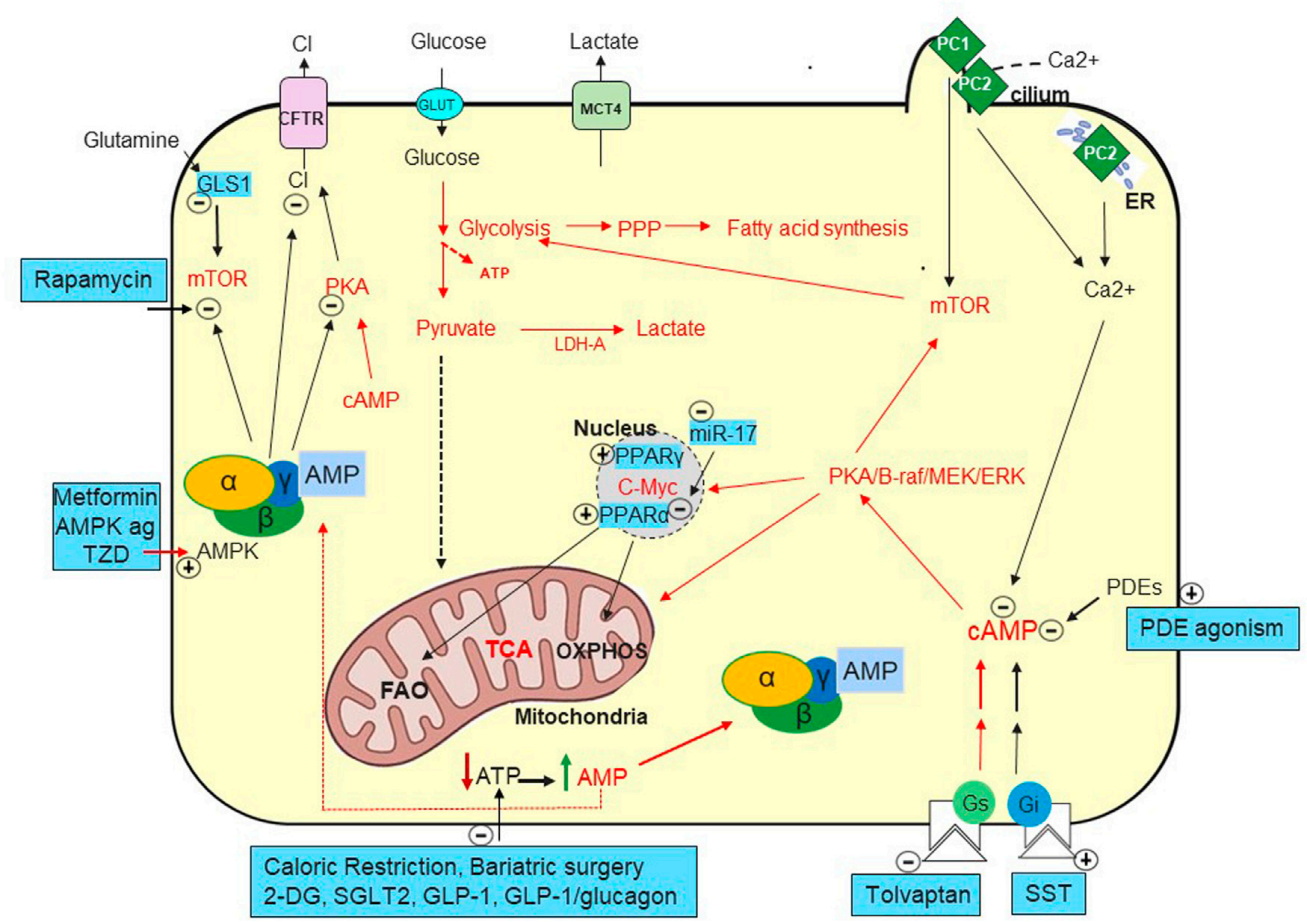
Metabolic Pathways in ADPKD. Increased cAMP due to vasopressin (Chebib et al., 2015) but likely also to decreased phosphodiesterases (PDEs) (Pinto et al., 2016) play a key role in generating fluid-filled cysts. Defects in PC1 and PC2 mediated calcium ion influx in the primary cilia and/or in the endoplasmic reticulum (ER) (Nauli et al., 2003; Padhy et al., 2022). Decreased intracellular calcium seems to convert the antiproliferative to proliferative effect of cAMP (Yamaguchi et al., 2003) causing activation of MEK-ERK and increased cell proliferation. cAMP activates PKA and stimulates chloride secretion through the cystic fibrosis transmembrane conductance regulator (CFTR) (Sullivan et al., 1998). Tolvaptan, a vasopressin V2 receptor antagonist, was approved to preserve kidney function by targeting cAMP (Chebib and Torres, 2021). Several somatostatin analogues (SST) are being investigated to lower cAMP with Ocreotide-LAR being approved in ADPKD in Italy (Capuano et al., 2022a). Mammalian target of rapamycin (mTOR) and c-Myc are upregulated in ADPKD and suppress oxidative metabolism while enhancing glycolytic flux, lactate production and export (LDH-A and MCT4) (Rowe et al., 2013; Podrini et al., 2020).

### Octreotide-LAR

Octreotide long-acting release (octreotide-LAR) is a somatostatin analogue that lowers the annual slope of TKV increase with no effect on renal function worsening. It was approved in Italy for the treatment of adult ADPKD patients at high risk of progression with eGFR ranging from 15 to 30 ml/min/1.73 m2 based on less frequent doubling of serum creatinine or ESKD compared to placebo. Side effects reported for somatostatin analogues include cholelithiasis and risk of cholecystitis, alopecia and increases in blood glucose (Perico et al., 2019; Griffiths et al., 2020). Hepatic cyst infections were also reported in patients treated with Lanreotide which required hospital admission and antibiotics. It has been suggested that the small size of the trials involving somatostatin analogues can explain the inconclusive renoprotective effects (Meijer et al., 2018; Capuano et al., 2022b). While awaiting publication of the results of Lanreotide in ADPKD (LIPS, NCT02127437) a plausible explanation for the apparent different effects across analogues is the affinity for the five somatostatin receptors (SSTR1 to 5) (Suwabe et al., 2021; Bais et al., 2022; Ruggenenti et al., 2022).

## Metabolic reprogramming in ADPKD

### Glutamine metabolism and aerobic glycolysis

The idea of metabolic reprogramming first came from the Warburg effect in cancer cells where OXPHOS is inhibited and cells tend to utilize aerobic glycolysis to produce energy (Koppenol et al., 2011). Metabolic reprogramming does not include only the Warburg effect, but also other metabolic changes. Rowe first suggested that mutations in Pkd1 result in a defective glucose metabolism with decreased gluconeogenesis and increased aerobic glycolysis to supply energy and promote proliferation (Rowe et al., 2013, Figure 1). 2-deoxyglucose (2DG), which is transported into the cells but cannot undergo glycolysis, inhibited the proliferation of Pkd1^−/−^ cells and prevented disease progression in ADKPD models (Rowe et al., 2013; Chiaravalli et al., 2016; Riwanto et al., 2016; Lian et al., 2019). Recently, Soomro provided evidence that alteration in glutamine metabolism play a role in cyst growth (Soomro et al., 2020). During glutaminolysis the enzyme glutaminase (GLS, Figure 1) converts glutamine to glutamate then converted to a TCA cycle intermediate, alpha-ketoglutarate to generate ATP for cyst growth (Soomro et al., 2020). Podrini confirmed the defective glucose metabolism and characterized other altered metabolic pathways in mouse kidney without Pkd1 such as increased pentose phosphate pathway (PPP), glutamine uptake and decreased TCA cycle and fatty acid oxidation (FAO) (Podrini et al., 2020). The authors also generated data supporting targeting asparagine synthetase to interfere with glutaminolysis in conjunction with glycolysis to slow PKD1^−/−^ cell growth and survival (Podrini et al., 2020). Decreased FAO also appears to contribute to disease exacerbation as increased c-MYC upregulates miR-17 in mouse cystic kidneys inhibiting PPARα and leading to FAO inhibition to support proliferation of ADPKD cells. Anti-miR-17 restored PPARα and improved FAO, ameliorating ADPKD (Lee et al., 2019, Figure 1). Considering that a single miRNA specie can regulate hundreds of targets, it is unclear if the beneficial effect is mediated by PPARα (Mohr and Mott, 2015). Nevertheless, the PPARα agonist fenofibrate showed increased FAO and reduced cystic volume in preclinical ADPKD (Lakhia et al., 2018). An anti-miR17 oligonucleotide is in Phase 1b in ADPKD patients to de-repress multiple miR-17 mRNA targets including Pkd1 and Pkd2 (Lakhia et al., 2022; https://www.prnewswire.com/news-releases/regulus-therapeutics-announces-first-patient-dosed-in-phase-1b-multiple-ascending-dose-mad-clinical-trial-of-rgls8429-for-the-treatment-of-autosomal-dominant-polycystic-kidney-disease-adpkd-301665896.html).

### Mammalian target of rapamycin (mTOR)

The mechanisms that account for elevated mTOR activity in ADPKD are not fully understood but it appears that cAMP/PKA/ERK and AKT are upstream regulators (Distefano et al., 2009; Rowe et al., 2013; Margaria et al., 2020). Animal studies demonstrate that mTOR inhibition improves cystic disease and kidney function (Pema et al., 2016; Su et al., 2022), however, metanalysis of cIinical data with ADPKD patients receiving rapamycin, sirolimus, or everolimus did not support a significant influence on renal progression (Lin et al., 2019). In these trials, it is not clear if mTOR inhibition was achieved in the kidney or whether mTORC1 inhibition triggers a compensatory activation of mTORC2 limiting the beneficial effects of mTORC1 (Canaud et al., 2010). Recently Janssen announced the acquisition of Anakuria Therapeutics and its first-in-class ADPKD candidate, AT-20494, a small molecule inhibitor of mTORC1 (https://www.fdanews.com/articles/206455-janssen-acquires-anakuria-therapeutics-nets-early-phase-polycystic-kidney-disease-candidate).

### AMP-activated protein kinase (AMPK)

AMPK is activated under conditions of metabolic and other cellular stresses (Steinberg and Hardie, 2022). AMPK activation during low energy states leads to upregulation of energy generating processes and inhibition of energy-intensive processes involved in cyst expansion such as indirect inactivation of mTORC1 (Inoki et al., 2003; Gwinn et al., 2008) and inhibition of CFTR chloride channel, thus suppressing epithelial fluid and electrolyte secretion (Caplan, 2022).

### Metformin

Metformin, a drug approved for T2D and polycystic ovary syndrome, may serve as a therapy for ADPKD. Treatment of kidney epithelial cells leads to stimulation of AMPK and subsequent inhibition of both mTOR and CFTR (Takiar et al., 2011). However, mixed results in animal models of PKD (Takiar et al., 2011; Leonhard et al., 2019; Lian et al., 2019; Chang et al., 2022; Pastor-Soler et al., 2022) and increased plasma lactate levels observed in Pkd1 miRNA transgenic mice, call for a careful examination of the risk benefit of metformin especially in patients with later stage of ADPKD (Chang et al., 2022). The Trial of administration of Metformin in PKD (TAME PKD, NCT02656017; Seliger et al., 2020) in 97 non-diabetic ADPKD adults with eGFR>50 ml/min per 1.73 m^2^ (Ong and Gansevoort, 2021; Perrone et al., 2021) suggests that metformin is safe in patients in the early stages of ADPKD (although only 35% completed the study at the maximal dose resulting in dose reductions). Results of the exploratory secondary endpoints were, however, inconclusive, with non-significant trends for eGFR slope and htTKV. A definitive answer should come from the IMPEDE PKD trial where a slow-release formulation of 2000 mg/d Metformin will be tested in 1164 patients with rapid progressive ADPKD over 2 years with estimated completion in 2026 (NCT04939935).

### Direct AMPK activation

While metformin inhibits renal cyst growth in mouse models, it remains unclear whether its metabolic effects are related to its capacity to activate AMPK (Takiar et al., 2011; Chang A. R. et al., 2017), and it may have tolerability issues. Hence potent selective AMPK activation may be required in ADPKD. Recently, the AMPK activator PF-06409577 demonstrated inhibition of mTOR pathway-mediated proliferation of cyst-lining epithelial cells and reduced CFTR-regulated cystic fluid secretion (Su et al., 2022). Given the potential for cardiac hypertrophy of AMPK following chronic administration it will be important to define the precise isoform selectivity required (Myers et al., 2017). Recently the FDA granted Orphan Drug Designation to Poxel’s AMPK activator PXL770 for the treatment of patients with ADPKD (https://www.poxelpharma.com/en_us/news-media/press-releases/detail/224/poxel-announces-pxl770-granted-orphan-drug-designation-from).

## Cholesterol reducing agents

### Statins

Statins (HMG-CoA reductase inhibitors) are widely prescribed to lower cholesterol in humans (Ginsberg and Tuck, 2001). Among the additional effects that make statins attractive for use in ADPKD (Belibi and Edelstein, 2010) is activation of AMPK (Sun et al., 2006) and cAMP lowering (Kou et al., 2012). Statins have been shown to improve early-onset ADPKD (TKV improvement) in children and young adults (Cadnapaphornchai et al., 2014). Recently, Baliga conducted targeted metabolomics in plasma samples from a phase III trial designed to test the efficacy of pravastatin on ADPKD progression in children and young adults on the ACE inhibitor (Baliga et al., 2021). The authors demonstrated changes in metabolites involved in metabolic reprogramming however statin treatment for 36 months had limited effect on disease progression (Baliga et al., 2021). While these results are overall encouraging, a larger randomized trial in young people with ADPKD is required. In the absence of such data, no consensus was reached on the use of statins in this population (Gimpel et al., 2019). An ongoing trial evaluating 2 years treatment with pravastatin in 150 adults with early stage ADPKD (NCT03273413) should also clarify the s inconclusive results in adult ADPKD (van Dijk et al., 2001; Fassett et al., 2010; Brosnahan et al., 2017; Xue et al., 2020).

### Bempedoic acid

Bempedoic acid (BA) antagonizes the ATP citrate-lyase (ACLY) enzyme upstream of HMGCoA reductase and is approved as an adjunct to diet and statin therapy in familial hypercholesterolemic patients who require additional lowering of LDL-C (Huynh, 2019; Ruscica et al., 2022). In animal models BA also activates AMPK (Pinkosky et al., 2016; Hallows et al., 2022) and reduces cystic growth, TKW and BUN (Hallows et al., 2022). BA seems to have a reduced risk of muscle-related side effects reported with statins (Ruscica et al., 2022) although a recent meta-analysis concluded that statins cause a small risk of muscle symptoms that are outweighed by the known cardiovascular benefits of statins (Cholesterol Treatment Trialists' Collaboration, 2022). No major safety concerns were identified for BA in a randomized controlled phase III trial during the intervention period when added to statin therapy, but the incidence of AEs leading to discontinuation was higher in the BA group, as was the incidence of gout (Ray et al., 2019). Bempedoic acid was generally well-tolerated following a single oral dose in subjects with renal impairment (Amore et al., 2022). Because ACLY has been reported to inhibit the AMPK-β1 subunit (Lee et al., 2015), future studies should conclusively demonstrate that the beneficial effects of BA in ADPKD are mediated by AMPK activation and clarify the AMPK subunit involved.

### Weight loss and insulin resistance

Similar to the general population, the prevalence of overweight and obese ADPKD patients is increasing. In rodent models of ADPKD, caloric restriction has shown to slow kidney growth and improve kidney function (Kipp et al., 2016; Warner et al., 2016). It has been suggested that these improvements involve mTOR signaling inhibition, AMPK activation, and a reduction in IGF-I supporting restoration of metabolic reprogramming. Accordingly, a clinical trial evaluating the effect of weekly caloric reduction achieved with either caloric restriction or intermittent fasting in 29 overweight/obese individuals with ADPKD was recently completed (Hopp et al., 2021; https://clinicaltrials.gov/ct2/show/NCT03342742). The trial was designed as a weight loss intervention based on the prior epidemiological observation that ADPKD progression is faster with higher BMI (Nowak et al., 2018). The investigators demonstrated the feasibility of 1-year daily caloric restriction (DCR) and intermittent fasting (IMF) in a cohort of overweight or obese patients with ADPKD. Weight loss occurred with both DCR and IMF, however, weight loss was greater, and adherence and tolerability were better with caloric restriction (Hopp et al., 2021). The study was a pilot and feasibility study, so the sample size was small, and a control group was not included but, according to the investigators, similar annual kidney growth in both groups was observed that was qualitatively low compared to historical controls. Cessation of kidney growth was observed in participants who achieved clinically meaningful weight loss (Nowak et al., 2018; Nowak et al., 2021). A larger 2-year phase 2 trial with a direct comparison of caloric restriction to a control group, powered for a primary endpoint of change in htTKV (NCT04907799) is currently recruiting with an expected completion in 2026 (https://clinicaltrials.gov/ct2/show/NCT04907799).

### Ketogenic diet

Ketosis improves the phenotype of animal models of ADPKD (Torres et al., 2019b). From a mechanism of action perspective, it is possible that Ketone bodies may promote metabolic reprogramming by decreasing glucose availability and increasing fatty acids (Hall et al., 2016). Initial data from clinical trials are becoming available. A self-enrolled survey of ADPKD patients who have self-administered ketogenic diet for at least 6 months reported weight loss and blood pressure lowering together with improvement in PKD symptoms and eGFR in a subgroup of patients. Caution should be applied to the interpretation of this retrospective study since only half of the patients were able to comply with the diet and the side effects reported suggest potential long-term tolerability and safety issues (kidney stones, increased cholesterol) (Strubl et al., 2021). In a follow-up study, Oehm demonstrated the feasibility of a short-term ketogenic intervention in 10 ADPKD patients (RESET-PKD 72h fast or 14 days of a KD) where TLV was decreased while no changes in TKV were observed (Oehm et al., 2022a; Oehm et al., 2022b). Despite the challenges identified, large-scale trials such as the ongoing KETO-ADPKD (NCT04680780) study will address the feasibility and the therapeutic potential of longer-term ketogenesis interventions in ADPKD (Ong and Torra, 2022). Adherence (ketone concentrations), feasibility and secondary outcomes including TKV, and BMI will be evaluated. Based on these findings, Bruen and collaborators have designed a plant-focused ketogenic diet (Ren.Nu diet) for ADPKD based on the theory that a diet high in carbohydrate and animal protein might accelerate disease progression (Bruen et al., 2022). A preliminary beta test was conducted for 12 weeks in 24 ADPKD patients and with the obvious limitations of the study (no control, selection bias, self-reporting). Preliminary data suggest reasonable adherence and feasibility (Bruen et al., 2022).

### Bariatric surgery

Bariatric surgery is an effective option to achieve sustained weight loss and improving hypertension and diabetes. Gastric bypass and sleeve gastrectomy result in 20%–30% weight loss (Brajcich and Hungness, 2020) and is expected to impact metabolic reprogramming. Therefore, it is important to understand the benefits and risks of bariatric surgery in ADPKD patients. While evidence suggests important trends for bariatric surgery and overall kidney related outcomes in patients with CKD, there exist several renal risks, including acute kidney injury, and risks of nephrolithiasis, oxalate nephropathy that will need to be considered in a comprehensive risk benefit assessment profile in ADPKD patients (Chang M. Y. et al., 2017).

### Glucagon-Like Peptide-1 (GLP-1) receptor agonism

Several GLP-1 receptor agonists have been approved for the treatment of T2D and obesity and are being considered for liver and kidney complications (Müller et al., 2019; Brown et al., 2021; Newsome et al., 2021). One attractive feature of new generation GLP-1 analogues is the propound weight loss (>10%) achieved in obese and diabetic patients (Frías et al., 2021). Importantly, GLP-1 exerts its effects by binding to GLP-1R and activating adenylate cyclase, which leads to the generation of cAMP, so it will be important to assess the expression of GLP-1R on the cystic epithelium and the potential impact of GLP-1 agonism on cAMP (Körner et al., 2007; Müller et al., 2019).

#### GLP-1R/glucagon receptors dual agonism

Dual agonism at the GLP-1 and glucagon receptors has shown superior weight lowering effect to selective GLP-1 agonism (Pocai et al., 2009; Day et al., 2012). Because glucagon lowers mTORC1 and stimulates AMPK (Baum et al., 2009; Welles et al., 2020) and ketogenesis (Pocai, 2012; Torres et al., 2019a), the simultaneous agonism of GLP-1R and glucagon receptors constitute a potential approach for ADPKD. A recent observational study in ADPKD patients with higher endogenous glucagon did not provide evidence for a protective role of glucagon in ADPKD (Knol et al., 2021). Mechanistic studies are needed to determine the relationship between glucagon and ADPKD and evaluate the expression of the receptor in kidney cysts. Future studies will clarify if the body weight lowering effect together with the other reported desirable actions of GLP-1 agonists have potential in ADPKD.

### Thiazolidinediones (TZD)

TZD are Peroxisome Proliferator Activator Receptor gamma (PPARγ) agonists approved for T2D. Preclinical studies have tested TZD and found reduced progression of cystic disease (Blazer-Yost et al., 2010; Flaig et al., 2016). A small phase 1b clinical trial was designed to investigate safety and tolerability of low-dose (15 mg) pioglitazone (Blazer-Yost et al., 2021). Concerns about fluid retention, bone loss and weight gain have reduced their use in the clinic and need to be considered in an appropriate risk/benefit assessment in ADPKD patients (Lebovitz, 2019).

### Sodium–glucose cotransporter (SGLT2) inhibition

SGLT2 inhibitors (SGLT2i) prevent the reabsorption of filtered glucose from the tubular lumen resulting in glucose lowering and additional benefits of weight loss and blood pressure reduction (DeFronzo et al., 2021). While the mechanisms contributing to these beneficial effects are unknown, SGLT2i switch metabolism to a ketotic state and increase plasma glucagon potentially regulating PKA and mTOR pathways (Saponaro et al., 2018). ADPKD patients are more prone to urinary tract infections and were excluded from renal studies as genitourinary infection is a potential risk of SGLT2i (Afsar et al., 2022). In two PKD animal models, SGLT2 inhibition did not reduce cyst growth (Kapoor et al., 2015; Rodriguez et al., 2015). However, because of the beneficial effects of SGLT2 inhibition on kidney function, vascular function, and mortality (DeFronzo et al., 2021), a clinical trial is ongoing in ADPKD patients (NCT05510115).

## Discussion

ADPKD is the leading genetic cause of ESKD. Recent advances in understanding the mechanisms leading to cyst formation and progression has led to the approval of tolvaptan (Torres et al., 2017). Treatment of ADPKD still represents a challenge due to the poor tolerability and the unfavorable safety profile of Tolvaptan. Thanks to new scientific discovery and preclinical models, new targets are being investigated. Metabolic defect in ADPKD support cell proliferation of rapid growing tissues leads to cystic epithelial proliferation and growth. Upregulated mTOR and c-Myc play a major role in repressing oxidative metabolism and FAO while enhancing glycolytic flux, lactic acid production, PPP and glutaminolysis downstream of cAMP/PKA (Rowe et al., 2013; Podrini et al., 2020). It will be important to expand upon the role of dysregulated metabolism as metabolic defects in cells within the kidney microenvironment may also contribute to ADPKD progression. A better understanding of the human pathways regulated by approved therapies (Tolvaptan and Octreotide-LAR) and initial results from ongoing trials with metabolic drugs should provide valuable human data and help expedite the development of new ADPKD therapeutics. Recently, the trial in ADPKD with Venglustat (Glucosylceramide synthase inhibitor; Natoli et al., 2010) was discontinued as it did not reduce TKV growth rate (https://www.sanofi.com/en/media-room/press-releases/2021/2021-06-01-05-00-00-2239122). Trial testing metformin in ADPKD should be completed by 2026 and provide valuable information (Testa and Magistroni, 2020). Weight loss targeting metabolic alterations has the potential to be a disease modifying intervention in ADPKD, but behavioral dietary interventions are limited by long-term adherence (Quiroga and Torra, 2022) so it will be important to explore the potential of bariatric surgery and pharmacological approaches. Therefore, it is tempting to speculate that interventions targeting downstream events such as metabolic reprogramming may retain and improve on the tolerability and efficacy reported with Tolvaptan offering potential new therapeutic opportunities. Ongoing trials may help in answering some of these questions.
